# Effects on Force, Velocity, Power, and Muscle Activation of Resistances with Variable Inertia Generated by Programmable Electromechanical Motors During Explosive Chest Press Exercises

**DOI:** 10.3390/bioengineering12030292

**Published:** 2025-03-14

**Authors:** Luca Zoffoli, Silvano Zanuso, Andrea Biscarini

**Affiliations:** 1 Scientific Research and Innovation Department, Technogym S.p.A., 47521 Cesena, Italy; lzoffoli@technogym.com; 2 School of Medical and Health Sciences, Edith Cowan University, Joondalup, WA 6027, Australia; 3 Department of Medicine and Surgery, University of Perugia, 06132 Perugia, Italy; andrea.biscarini@unipg.it

**Keywords:** resistance training, variable inertia, power, EMG

## Abstract

Strength training machines incorporating advanced electro-mechanical technologies can produce hybrid resistances with variable inertia, such as a resistance that progressively changes from gravitational (inertial) to pneumatic (non-inertial) across the range of motion (ROM). To explore the biomechanical effects of these innovative resistances, a robotic chest press machine was programmed to offer three distinct inertial profiles: gravitational-type constant inertia throughout the ROM (I_FULL_); no inertia (I_ZERO_); and linearly descending inertia across the ROM (I_VAR_). Ten healthy adults performed five maximal-effort, explosive chest press movements under each inertial profile at 30, 50 and 70% of their one-repetition maximum. During each trial, muscle activity of the pectoralis major, anterior deltoid, and triceps brachii was recorded, along with force, velocity and power outputs from the machine. Statistical non-parametric maps based on two-way repeated measures ANOVA were used to assess the effects of load level and inertial profile on the collected time series. Higher load levels consistently led to increased force and reduced velocity and power outcomes over large parts of the ROM. Compared to I_FULL_, I_ZERO_ allowed for greater velocity at the expense of lower force throughout the ROM, while I_VAR_ produced higher force and power outputs despite having lower velocity than I_ZERO_. Additionally, both I_ZERO_ and I_VAR_ significantly increased triceps brachii activity at the end of the ROM compared to I_FULL_. I_VAR_ outperformed both I_FULL_ and I_ZERO_ in terms of force and power. Coaches and therapists are advised to consider variable inertial profiles as a key parameter when designing exercise programs for athletes or patients.

## 1. Introduction

Resistance training is a form of physical exercise designed to improve muscular strength, endurance, power, and hypertrophy by exercising a muscle or muscle group against external resistance [1]. External resistance can take the form of free weights (e.g., dumbbells, barbells), elastic bands, weight machines, pneumatic devices, or other objects that oppose muscle contraction [2]. Although various forms of resistance have been studied [2], the widespread availability of free weights, weight-stack machines, and plate-loaded machines, which offer a wide range of constant and variable resistances, has led to the extensive use of gravitational resistance in sports [3], fitness [4], and rehabilitation [5]. Free weights are generally thought to provide constant resistance throughout the movement, while weight-stack machines, through specifically designed asymmetric pulleys, are considered to produce isotonic variable-resistance profiles [6]. However, both concepts can be misleading, as they overlook the contribution of inertial force to the overall resistance opposing the movement.

The inertial force acting on a free weight or a weight stack lifted vertically is determined by the product of the lifted mass m and its acceleration $\vec{a}$ [7]. In the case of weight machines, inertial effects also include the product of the moment of inertia and the angular acceleration of rotating elements, such as resistance levers, cams, and pulleys [7]. Thus, inertial forces become particularly relevant in exercises involving gravitational resistance and substantial acceleration or deceleration phases. This is especially true in maximal-effort lifts with submaximal weights aimed at developing muscle power and high-velocity strength [8].

To understand the biomechanical effects of inertial forces, it is necessary to apply basic physics to a free weight lifted in the vertical direction. At the beginning of the lift, the magnitude F of the vertical force $\vec{F}$ applied to resistance mass *m* must overcome the magnitude mg of the gravitational force. The surplus F−mg generates an acceleration $\vec{a}$ with magnitude a determined by Newton’s law: ma=F−mg. Therefore, the user provides a force F=mg+ma, which equals the sum of mg and the magnitude ma of the inertial force $m\vec{a}$ needed to produce the acceleration $\vec{a}$. During this phase, the resistance mass progressively gains velocity $\vec{v}$ and momentum $m\vec{v}$ [9,10]. Conversely, during the final phase of the movement, the user must intentionally apply a force with a magnitude F lower than mg to decelerate the resistance mass and bring it to a stop by the end of the lift. According to Newton’s law, ma=mg−F, the deceleration magnitude a is proportional to the difference mg−F. In this phase, the user applies a submaximal force, lower than mg by an amount equal to the magnitude of the inertial force $m\vec{a}$ needed to produce the load deceleration $\vec{a}$ (F=mg−ma) [9,11]. In ballistic exercises (such as the bench throw and shot put), the load is accelerated throughout the range of motion (ROM) and released into free space at the end of the movement [12]. However, these exercises require specific equipment and safety precautions, or the availability of large spaces for the projection of implements [12].

Inertial effects have significant consequences on both exercise safety and adaptations. The light gravitational loads used for high-velocity strength display a deceleration phase occurring over a substantial portion (up to 40%) of the final part of the ROM [11]. Attempting to accelerate the load until almost the end of the ROM can transfer the load momentum to the joint tissues, potentially leading to excessive joint loading [13]. Consequently, peak velocity and peak power are reduced, and the training stimulus is compromised [14]. This limitation is addressed by using strength machines with minimal moving masses, such as those employing pneumatic or elastic resistance [15,16]. However, the mass of the equipment or the athlete’s own body weight plays a critical role in the initial phase of explosive sports movements, where a sharp acceleration peak generates a sudden burst of inertial force (the product of the mass and its acceleration) [7]. As a result, resistance systems with negligible inertial mass, such as pneumatic or elastic systems, provides a less specific training stimulus during the initial stages of explosive movements and may significantly reduce both the rate of force development (RFD) and peak force output [9,17]. In fact, applying explosive force against non-inertial resistance leads to a rapid increase in velocity at the start of the movement [7], which in turn, decreases muscle force due to the force–velocity relationship [18]. Theoretical models suggest that the development of key muscle power components (RFD, high-velocity strength and maximal force [12]) during explosive exercises could be optimized with the use of inertial resistance during the initial phase of the movement and non-inertial resistance in the final phase [9].

Previous experimental research on the effects of inertial forces during resistance exercise has largely been limited to comparing gravitational, flywheel, pneumatic, and elastic resistances [15,19,20,21,22,23]. Furthermore, these resistances were produced by different exercise apparatuses, making it difficult to isolate the inertial effects from those caused by the specific mechanics of the equipment (resistance profile, constraints on movement trajectory, stabilization supports, etc.). Some studies have examined the effects of the simultaneous application of resistances with different inertial properties, such as gravitational and pneumatic [17] or gravitational and elastic [24,25,26], combining these resistances in different proportions. Modern strength training machines equipped with advanced electromechanical technologies now enable the generation of hybrid resistances with variable inertia, providing resistance that transitions suddenly or progressively from inertial (e.g., gravitational) to non-inertial (e.g., pneumatic) across the range of motion [9]. This novel resistance is theorized to combine the advantages of both inertial and non-inertial resistance while minimizing their limitations, optimizing force, velocity and power during explosive exercises [9]. However, there is lack of experimental data on the effects of this new training paradigm, known as variable inertia training (VIT) [9].

Given this absence of experimental data and the opportunities presented by advanced technologies, this study aims to characterize the biomechanical effects of resistance with variable inertia in power-oriented training. To achieve this, a robotic chest press machine was programmed to provide three distinct inertial profiles: gravitational-type constant inertia throughout the ROM (I_FULL_), no inertia (I_ZERO_), and linearly descending inertia across the ROM (I_VAR_). Force, velocity and power outputs and muscle activation of prime movers were recorded during maximal-effort, explosive exercises executed under each inertial profile and different intensities (30, 50 and 70% of the one-repetition maximum). The results indicate that the I_VAR_ profile, which features linearly decreasing inertia, generally produces superior outcomes in terms of performance metrics compared to both I_FULL_ and I_ZERO_. Based on these findings, strength and conditioning professionals, as well as rehabilitation specialists, are encouraged to consider variable inertial profiles as a key parameter when designing training programs for athletes and patients.

## 2. Materials and Methods

### 2.1. Subjects

Ten healthy males (age: 41.8 ± 10.7 years; weight: 83.8 ± 5.8 kg; height: 1.79 ± 0.09 m) with advanced experience in resistance training were voluntarily recruited from local gyms and fitness centers. The eligibility criteria for this study were as follows: age between 18 and 64 years, current participation in sports activities, possession of a valid medical certificate for sports, and more than 3 years of structured and consistent resistance training experience with a minimum of 3 practice sessions per week. The exclusion criteria included musculoskeletal injuries, a history of limb or trunk pathologies, and the inability to perform the explosive chest press exercise without experiencing pain and while maintaining proper technique. The study adhered to the ethical guidelines and international standards set forth in the Declaration of Helsinki and was approved by the Ethics Committee of the University of Perugia. All participants provided written informed consent for inclusion in the study after being fully informed of the study’s purpose, experimental protocol, potential risks, and privacy considerations.

### 2.2. Exercise Equipment

**Figure 1:**
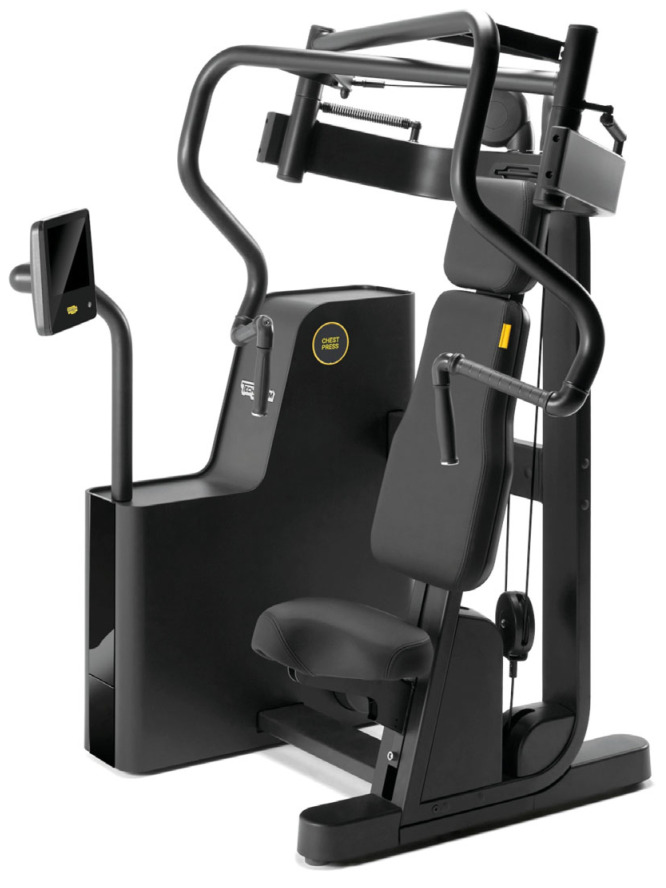
Exercise apparatus: Technogym Biostrength™ Chest Press (Cesena, Italy).

A commercially available, electro-mechanically driven exercise apparatus (Biostrength™ Chest Press, Technogym SPA, Cesena, Italy) was utilized (Figure 1). This type of equipment has been previously employed in research studies [27] and was selected for its unique capability to generate arbitrary resistance profiles, hybrid resistances that can change nature—for example, from gravitational to pneumatic—across the ROM, and novel resistance types incorporating an inertial component that varies arbitrarily, either in a pre-planned or adaptive mode, throughout the ROM. Furthermore, the apparatus records force and displacement data during the exercise at a sampling rate of 20 Hz with 16-bit resolution. Specifically, dynamic force measurements are obtained through a sophisticated servomechanism specifically designed to determine the difference between the resistance generated by the motor (which is known) and the force exerted by the user on the resistance levers. This is one the most important and innovative aspects of the technology used in Biostrength™ machines. The exercise apparatus also includes an embedded automatic calibration procedure provided by the manufacturer, which runs at each start or restart. This calibration process is operator-independent, ensuring accurate readings of the user’s ROM and generated force without requiring specific manual intervention.

### 2.3. Programmed Inertial Profiles

In general, the resistance offered by weight-stack machines can be described as follows [21]:

(1) $$\begin{array}{r} R\left(m,x,\ddot{x}\right)=S\left(m,x,\ddot{x}\right)+L\left(x,\dot{x},\ddot{x}\right) \end{array}$$

This equation expresses the resistive force *R* as function of the selected resistance mass (m), its position (x), and its acceleration ($\ddot{x}$). The right-hand side shows that R arises from the additive contribution of S, representing the weight stack, and *L*, which accounts for other moving parts of the apparatus (e.g., levers, cables, pulleys). More specifically, the contribution of S can be expressed as follows:

(2) $$\begin{array}{r} S\left(m,x,\ddot{x}\right)=K\left(x\right)\cdot \left(mg+m\ddot{x}\right) \end{array}$$

where g is the acceleration of gravity, and K(x) is a positive function representing the action of an asymmetric pulley typically used to produce an isotonic resistance profile. In contrast, the precise description of L is often complex, as it depends heavily on the design of the machine [28]. However, under standard exercise conditions, the contribution of L to F is typically orders of magnitude smaller than that of S. Consequently, *L* can often be neglected or approximated as a constant. Assuming K to be constant and independent of x (as in the case of free weights), the effective value of S (and therefore R) across the ROM is only modulated by the weight stack’s acceleration or deceleration. Specifically, compared to a quasi-static lift, S increases as the weight stack accelerates during the initial stage of the concentric phase, and decreases as the weight stack decelerates during the final stage of the concentric phase. To evaluate the effects of manipulating inertial force ($m\ddot{x}$) on force, power, and activation level of the prime movers, the equation for *S* was modified as follows:

(3) $$\begin{array}{r} S\left(m,x,\ddot{x}\right)=mg+\lambda \left(x\right)\cdot m\ddot{x} \end{array}$$

This modified equation introduces the function $\lambda \left(x\right)$, which modulates the inertial component $m\ddot{x}$ of the resistance based on the instantaneous position x, while leaving the gravitational component mg unchanged. Additionally, the K(x) function, which defines the isotonic cam profile of the apparatus, was set to K=1. This adjustment rendered the resistance profile like that of a standard free-weight exercise, thereby eliminating potential confounding effects of isotonic cams when assessing the effects of $\Lambda \left(x\right)$ on force, power and muscle activity. The three inertial profiles investigated in this study —gravitational-type constant inertia throughout the ROM (I_FULL_), no inertia (I_ZERO_), linearly descending inertia across the ROM (I_VAR_)—were defined by specific λ function as follows:

(4) $$\lambda_{I_{FULL}}\equiv 1$$

(5) $$\lambda_{I_{ZERO}}\equiv 0$$

(6) $$\lambda_{I_{VAR}}\left(ROM_{\%}\right)=\left\{\begin{array}{ll} 1 & ROM_{\%}\leq 25\\ 0 & ROM_{\%}\geq 75\\ 1.5-0.02\cdot ROM_{\%} & 25<ROM_{\%}<75 \end{array}\right.$$

Here, ROM_%_ refers to the instantaneous position of the resistance levers, expressed as a percentage (0–100%) of the individual range of motion determined by the exercise apparatus for each participant. Specifically, 0 ROM_%_ corresponds to the position of the resistance lever at the start of the concentric phase of the movement, while 100 ROM_%_ represents its final position at the end of this phase. Ultimately, this ROM% definition allows for the identification of any given lever position as a specific position within the complete range of lever positions utilized by each participant during the trials. As follows from Equation (3), $I_{FULL}$, defined by the condition λ=1, corresponds to the inertial profile of a conventional weight stack $mg+m\ddot{x}$, where resistance includes both gravitational (mg) and inertial ($m\ddot{x}$) components. I_ZERO_, defined by the condition λ=0, represents the idealized condition of complete absence of inertia ($m\ddot{x}=0$), with the resistance provided solely by the constant weight component *mg*. I_VAR_ describes a variable inertial profile that decreases linearly from I_FULL_ to I_ZERO_ across the 25–75% segment of the ROM of each individual participant.

### 2.4. Testing Procedure

Participants began with a general warm-up consisting of 5 min of cycling on a cycle ergometer (Skillbike™, Technogym SPA, Cesena, Italy) at a self-selected resistance. They then proceeded to sit on the exercise apparatus and adjust the seat height according to the manufacturer’s guidelines. Specifically, the center of their shoulder joints was aligned with a pair of yellow flags secured to the backrest. This setup ensured optimal body positioning for each participant, allowing for the efficient generation of force. The warm-up continued with three repetitions performed at the minimal load (10 kg) at a slow, constant pace. Each repetition lasted approximately 3 s for both the concentric and eccentric phases, with no isometric rest in between. Since the participants had advanced experience in resistance training, they were then allowed to complete their own specific warm-up phase to prepare for maximal pushing exercises.

After completing the warm-up, participants performed six repetitions with a self-selected load using the I_FULL_ resistance profile. Following a 3 min rest, the load was increased, and another six repetitions were performed. This procedure was repeated, typically 2–3 times, depending on the participant’s ability, until they could no longer complete six repetitions. The one-repetition maximum (1RM) was then estimated based on the load and the number of repetitions performed during the final set [29]. The progressive increase in load further prepared participants for maximal exertion. After an additional 3 min rest, participants completed three maximal isokinetic concentric-only repetitions. The movement speed was set to approximately 0.24 m/s (measured at the midpoints of the handles) to replicate the average lifting speed of a 1RM bench press [30].

Subsequently, participants performed nine sets of five repetitions at maximal concentric speed. These sets included all combinations of three inertial profiles (I_FULL_, I_ZERO_, I_VAR_) and three intensity levels (30%, 50% and 70% of 1RM, denoted as 1RM_30_, 1RM_50_, and 1RM_70_). Each set was separated by a minimum of 3 min of recovery, and the order of the inertial conditions was randomized for each participant. During the tests, participants were instructed to “push as much and as fast as possible”, and they were not aware of the specific inertial profile being applied.

### 2.5. Data Recording and Processing

Pairs of unipolar, Ag/AgCl, ⌀=24 mm, pre-gelled, disposable EMG electrodes (Covidien Kendall, Minneapolis, MN, USA) were secured to the right side of the body over the pectoralis major, anterior deltoid and the long head of the triceps brachii muscles [31]. These muscles were selected as they serve as prime movers for the studied exercise [32]. The electrodes were positioned parallel to the muscle fibers with an inter-electrode distance of 2 cm. Prior to placement, the skin was shaved, lightly abraded with sandpaper, and cleansed using an alcohol solution. EMG data (input impedance: 100 MΩ; CMRR: >>110 dB; baseline noise: <1 μV; gain: 240.6) were synchronously sampled at 1 kHz and recorded on a computer via a 16-bit resolution wireless system (FreeEMG, BTS Bioengineering SPA, Milano, Italy).

Pairs of semi-spherical reflective markers (⌀=10 mm) were attached to the ends of the exercise apparatus handles. Kinematic data were collected using a ten-camera motion capture system (SMART DX-7000, BTS Bioengineering SPA, Milano, Italy) synchronized with the EMG sensors. The data were sampled at 500 Hz with a 4 Mpixel resolution. System calibration yielded a measurement error smaller than 3 mm across all cameras.

Each EMG signal recorded from the right pectoralis major (EMG_PM_), anterior deltoid (EMG_DA_) and triceps brachii (EMG_TB_) muscles was centered around its mean and filtered using a fourth-order, band-pass, zero-phase Butterworth filter with a bandwidth of 20–450 Hz [31,33]. The signals were then full-wave rectified. For each trial, the *force* generated by the user over time was obtained from motor readings, while instantaneous *velocity* was determined by deriving the right handle midpoint position over time [34]. All data were subsequently processed with a linear envelope using a fourth-order, low-pass, phase-corrected Butterworth filter with cut-off frequency of 3 Hz [34]. *Force* data were time-synchronized with *velocity* and EMG data by detecting the time lag corresponding to the peak in the cross-correlation between the positional readings of the exercise apparatus and those of the right handle midpoint from kinematic acquisitions. *Power* output was then calculated as the product of *force* and *velocity*. The time instants corresponding to the beginning and end of the concentric phase of each repetition were identified by the following steps: (1) locating the local minima and maxima in the anterior–posterior displacement of the handles; and (2) identifying in the *velocity* signal the closest zero-crossing points to these minima and maxima. The first and last repetitions of each trial were excluded to avoid potential confounding effects from movement initiation or termination. For each muscle investigated, the processed EMG signals were normalized to the mean of the peak values obtained from repetitions performed with isokinetic resistance. Finally, for each detected concentric phase, the EMG_PM_, EMG_DA_, EMG_TB_, *force*, *velocity* and *power* data were interpolated using cubic splines to 101 samples.

### 2.6. Statistical Analysis

Because of the one-dimensional nature of the investigated data, statistical maps based on 2-way repeated measures ANOVA were generated to evaluate the effects of the lifted load and the inertial profile on each investigated parameter [35]. One-dimensional statistical mapping extends the traditional scalar (i.e., 0D) statistical testing procedure to time series. The theoretical foundation of such an approach is extensively described by other authors [35,36,37], but it can briefly be described as a statistical analysis that allows evaluation of the presence of regional differences in the time series according to distinct groups or covariates. During exploratory analysis, the best combination of terms in the model (*lifted load*, *inertial profile*, and their interaction) was evaluated by calculating Bayesian Information Criterion (BIC) [38] maps over each of the 101 samples, defining the time series of interest and then taking their median value. Lower BIC scores indicate better fit for a given model to the data as compared to a model with a higher BIC score. This analysis revealed that lower BIC scores were obtained by removing the interaction term (*lifted load x inertial profile*) from the analysis. Therefore, each parameter was analyzed considering only the main effects. The application of one-dimensional parametric statistical approaches to time series assumes the normal distribution of the data. This was evaluated by generating statistical Shapiro–Wilk test maps for each investigated parameter to evaluate the presence of regions in the time series where the normality assumption was violated. Violation of the assumption of normality was found for most of the investigated parameters. Hence, a nonparametric approach was employed to test the effect of the *lifted load* and *inertial profile* on the investigated parameters (*force*, *power*, *velocity*, EMG_PM_, EMG_DA_, and EMG_TB_). Permutation tests were used to detect regions of the ROM highlighted by significant differences caused by the distinct *lifted load or inertial profile*. The permutation test is a popular non-parametric technique that is usually employed in a 0D analysis that has already been successfully extended to one-dimensional tests [37]. When significant differences in specific regions of the ROM were found, the ROM_START-STOP_ notation was used to provide a concise representation of the significant regions emerging from the statistical analysis. $\eta^{2}$ maps were obtained to evaluate the effect size of the differences between the compared conditions. The mean $\eta^{2}$ ($\bar{\eta^{2}}$) was then calculated for each region of the ROM where significant differences were detected. Post hoc analyses were performed via permutation tests based on paired *t*-tests between the mean values obtained from the groups being compared over the significant region. For each post hoc comparison, the mean values with 95% confidence intervals of the compared quantities were reported. The level of significance (α) was set at 0.05, and 10.000 permutations were used to derive the distribution from which the *p*-values were extracted. The Holm–Sidak correction [39] was employed to adjust for multiple comparisons during post hoc analyses. All data were reduced, processed, and analyzed via custom Python scripts (version 3.11.3, https://www.python.org, accessed on 6 February 2025) using the libraries numpy (version 1.25, https://www.numpy.org, accessed on 6 February 2025), matplotlib (version 3.7.0, https://www.matplotlib.org), pandas (version 2.0.3, https://pandas.pydata.org, accessed on 6 February 2025), scipy (version 1.11.1, https://www.scipy.org, accessed on 6 February 2025), spm1d (version 0.4.18, https://www.spm1d.org, accessed on 6 February 2025), and statsmodels (version 0.14.0, https://www.statsmodels.org, accessed on 6 February 2025).

## 3. Results

**Figure 2:**
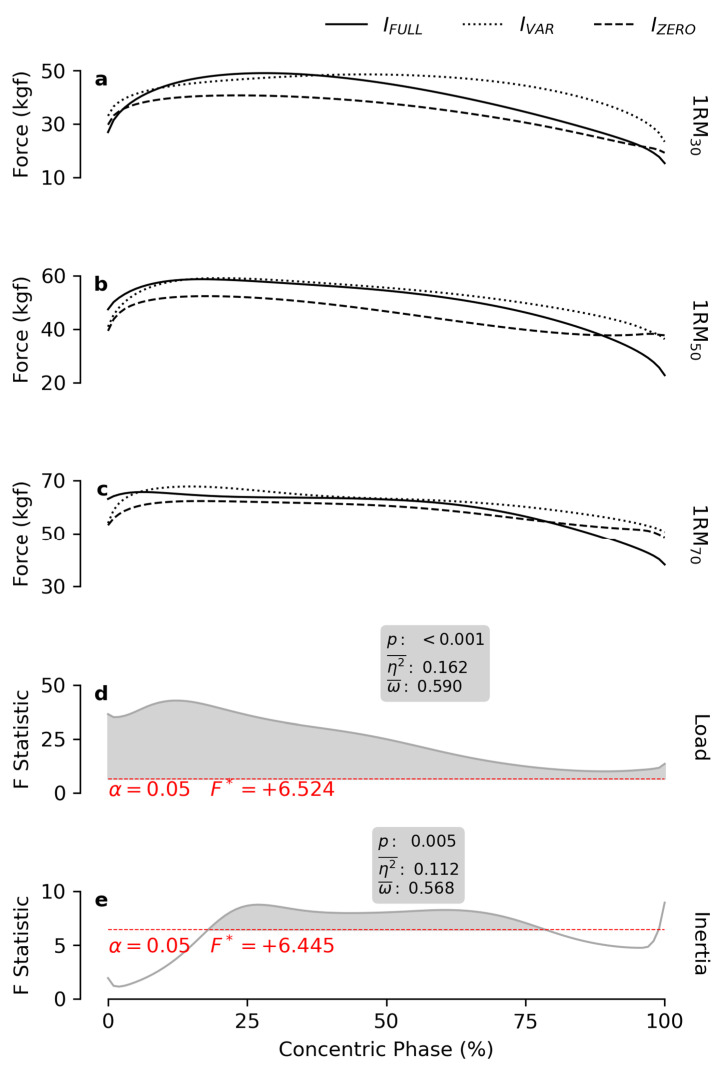
*Force* patterns. The subfigures (**a**–**c**) report the mean force patterns obtained at 30, 50 and 70% of the 1RM, respectively, for each inertial profile. The grey lines in subfigures (**d**,**e**) show the statistical maps for the *load* and *inertia* effects. The red dashes indicate the critical F value corresponding to α. The grey areas highlight the significant regions of the map, and the boxes report the calculated *p* values, effect size ($\bar{\eta^{2}}$), and observed power ($\bar{\omega }$).

*Force* increased with *load* across the entire ROM (Figure 2), with higher values observed at greater percentages of 1RM. In contrast, *inertia* influenced *force* production during the mid-range of the concentric phase (ROM_18–79%_). Post hoc analysis revealed progressively higher force values for I_ZERO_, I_FULL_ and I_VAR_, respectively (Table 1).

**Figure 3:**
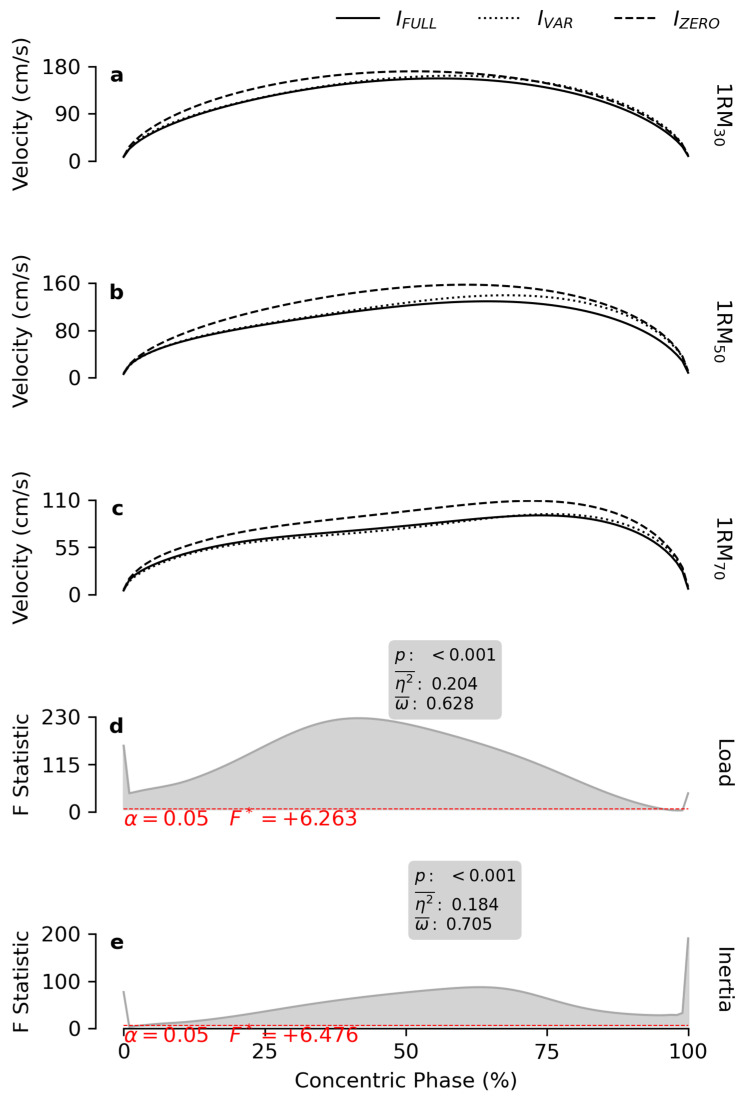
*Velocity* patterns. The subfigures (**a**–**c**) report the mean velocity patterns obtained at 30, 50 and 70% of the 1RM, respectively, for each inertial profile. The grey lines in subfigures (**d**,**e**) show the statistical maps for the *load* and *inertia* effects. The red dashes indicate the critical F value corresponding to α. The grey areas highlight the significant regions of the map, and the boxes display the calculated values of *p*, effect size ($\bar{\eta^{2}}$), and observed power ($\bar{\omega }$).

*Velocity* progressively decreased throughout most of the concentric phase (ROM_0–96%_) as *load* increased (Figure 3). Similarly, *inertia* significantly affected a large portion of the concentric phase (ROM_3–100%_), with progressively lower *velocity* values observed for I_ZERO_, I_VAR_ and I_FULL_, respectively (Table 1).

**Figure 4:**
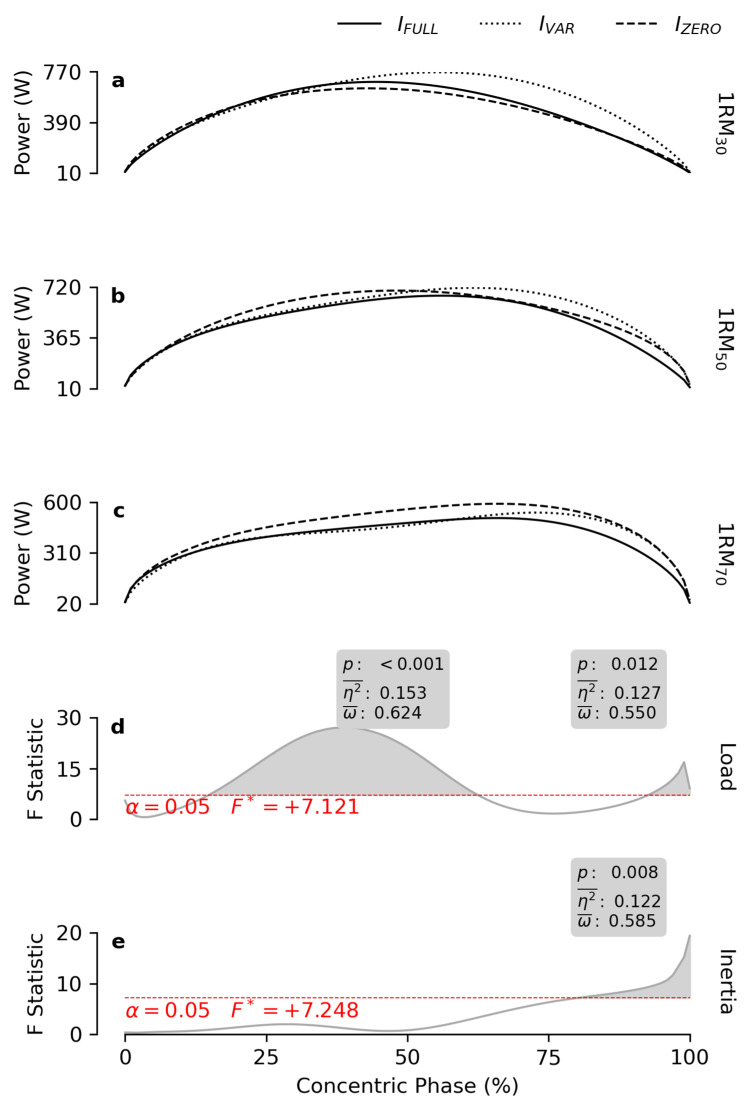
*Power* patterns. The subfigures (**a**–**c**) report the mean power patterns obtained at 30, 50 and 70% of the 1RM, respectively, for each inertial profile. The grey lines in subfigures (**d**,**e**) show the statistical maps for the *load* and *inertia* effects. The red dashes indicate the critical F value corresponding to α. The grey areas highlight the significant regions of the map, and the boxes display the calculated value of *p*, effect size ($\bar{\eta^{2}}$), and observed power ($\bar{\omega }$).

*Power* was influenced by *load* on two distinct ROM regions (Figure 4). For ROM_15–62%_, 1RM_70_ produced lower values than 1RM_50_ and 1RM_30_. Conversely, in the ROM_93–100%_ range, the opposite trend was observed, with 1RM_70_ showing the highest *power* compared to 1RM_30_ and 1RM_50_ (Table 1). *Power* was also affected by *inertia* at the end of the ROM (ROM_80–100%_), where *I_VAR_* achieved the highest values and I_FULL_ the lowest (Table 1).

**Figure 5:**
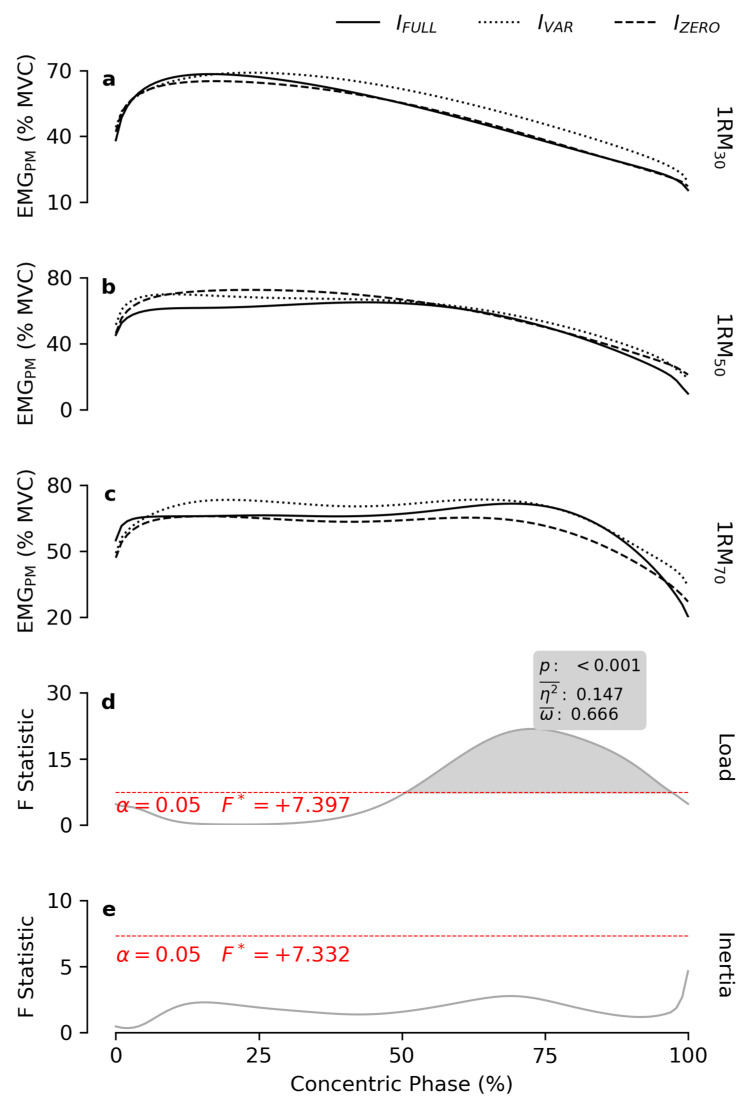
Normalized EMG_PM_ patterns. The subfigures (**a**–**c**) report the mean EMG patterns of the right pectoralis major muscle obtained at 30, 50 and 70% of the 1RM, respectively, for each inertial profile. The grey lines in subfigures (**d**,**e**) show the statistical maps for the *load* and *inertia* effects. The red dashes indicate the critical F value corresponding to α The grey areas highlight the significant regions of the map, and the boxes display the calculated value of *p*, effect size ($\bar{\eta^{2}}$), and observed power ($\bar{\omega }$).

EMG_PM_ amplitude was proportional to *load* (Figure 5) during the second half of the concentric phase (ROM_50–97%_). Conversely, *inertia* had no effect on any part of the ROM.

**Figure 6:**
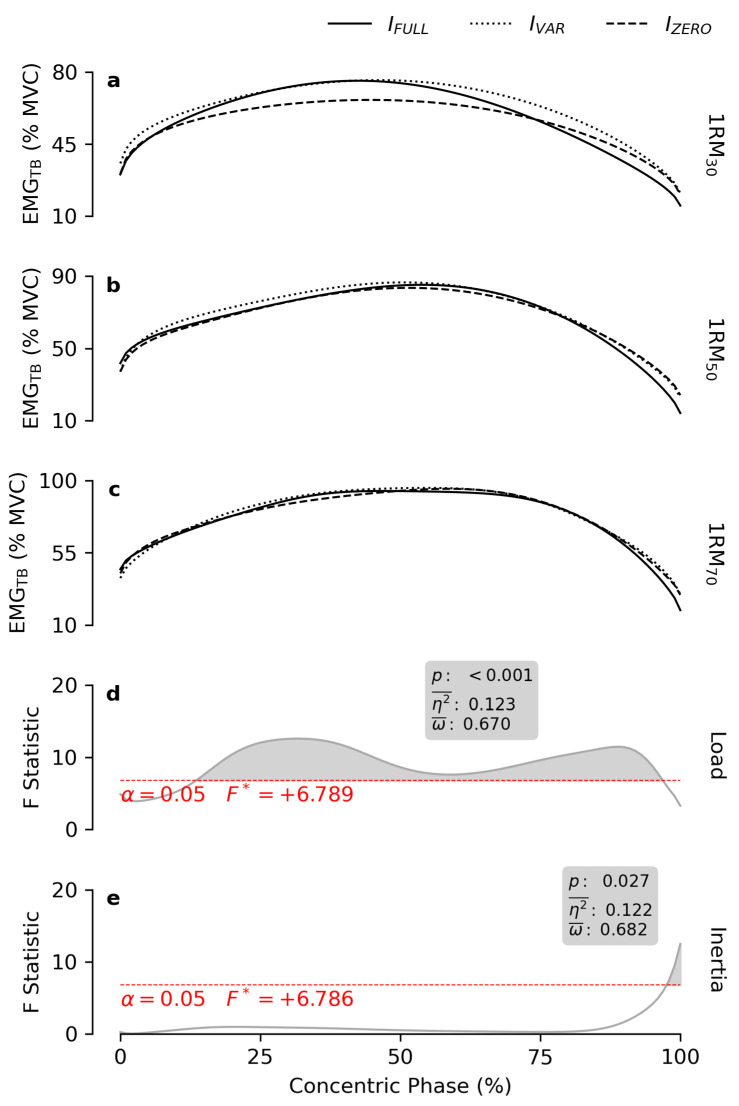
Normalized EMG_TB_ patterns. The subfigures (**a**–**c**) report the mean EMG patterns of the right triceps brachii muscle obtained at 30, 50 and 70% of the 1RM, respectively, for each inertial profile. The grey lines in subfigures (**d**,**e**) show the statistical maps for the *load* and *inertia* effects. The red dashes indicate the critical F value corresponding to α. The grey areas highlight the significant regions of the map, and the boxes display the calculated values of *p*, effect size ($\bar{\eta^{2}}$), and observed power ($\bar{\omega }$).

EMG_TB_ (Figure 6) was greater with higher loads throughout most of the ROM (ROM_14–97%_). Additionally, at the end of the concentric phase (ROM_98–100%_), I_FULL_ was reduced EMG_TB_ compared to I_ZERO_ and I_VAR_ (Table 1).

**Figure 7:**
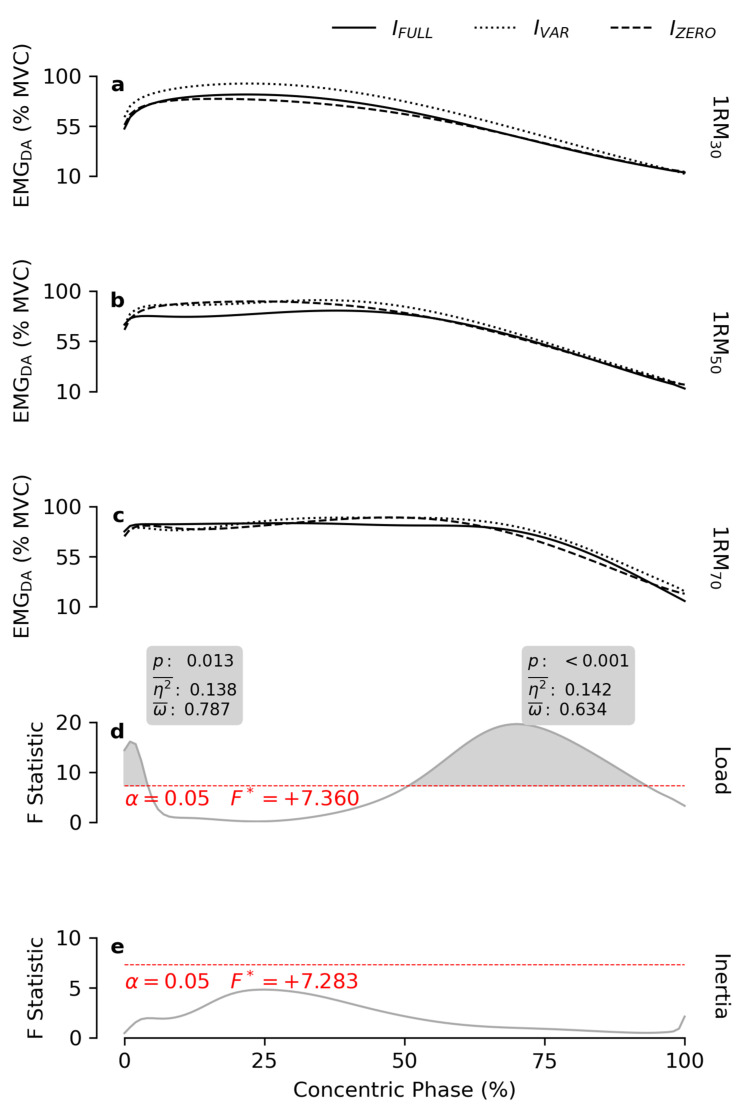
Normalized EMG_DA_ patterns. The subfigures (**a**–**c**) report the mean EMG patterns of the right anterior deltoid muscle obtained at 30, 50 and 70% of the 1RM, respectively, for each inertial profile. The grey lines in subfigures (**d**,**e**) show the statistical maps for the *load* and *inertia* effects. The red dashes indicate the critical F value corresponding to α. The grey areas highlight the significant regions of the map, and the boxes display the calculated values of *p*, effect size ($\bar{\eta^{2}}$), and observed power ($\bar{\omega }$).

EMG_DA_ was not influenced by *inertia*, although *load* showed significant effects in two regions of the ROM (Figure 7). For ROM_0–4%_, 1RM_30_ resulted in a lower EMG amplitude than 1RM_50_ and 1RM_70_, while at ROM_50–94%_, EMG_DA_ activity was proportional to *load* (Table 1).

## 4. Discussion

This study investigated the effects induced by different inertial resistance profiles on the force, velocity and power outputs as well as the activation levels of the pectoralis major, anterior deltoid and long head of the triceps brachii during a chest press exercise performed at different intensity levels.

In line with previous studies [40,41,42], higher intensity resulted in higher *force*. This effect was relevant across the whole ROM and was independent of the employed inertial profile. As predicted by theory [9], *force* was enhanced by the presence of inertial resistance at beginning of the ROM and reduced towards its end. Consequently, the absence of inertial resistance (as in the I_ZERO_ condition) limited *force* production, though it provided a flatter profile across the ROM. Notably, I_VAR_, which retained the inertial contribution of *I_FULL_* at the beginning of the ROM and gradually approximated the I_ZERO_ behavior toward the end of the concentric phase, maintained elevated *force* levels over a larger portion of the ROM and significantly outperformed both I_ZERO_ and I_FULL_ in the mid-range (Figure 2).

Consistent with previous findings [15], the inclusion of inertial resistance reduced *velocity* throughout the entire ROM (Figure 3). The same findings supported the use of *I_ZERO_* to achieve high *power* output. However, the present study highlights the importance of resistance with variable inertia in maintaining high power across the ROM. Notably, *power* output was unaffected by *inertia* during the first 80% of the ROM. Beyond this point, *power* progressively decreased across the three conditions I_VAR_, I_ZERO_ and I_FULL_ (Figure 4). This behavior likely reflects the dynamic interplay of *force* and *velocity*. When *I_FULL_* was used, the relatively low *velocity* was compensated for by higher *force* levels. However, the reduction in *force* induced by the facilitating effect of inertial resistance at the end of the concentric phase had the effect of limiting the *power* output compared to the other two conditions. Conversely, with I_ZERO_, the low *force* was offset by the highest *velocity* among the three conditions, enabling the production of significant *power* levels across most of the ROM. Nonetheless, the superior *power* achieved by I_VAR_ during the last 20% of the ROM suggests that the limited *force* produced with I_ZERO_ might have limited *power* output in the later portion of the ROM. Thus, while I_ZERO_ was most effective for eliciting high *velocity*, the variable inertia profile within the ROM defined by I_VAR_ appeared to combine the positive effects of I_FULL_ on *force* with those provided by I_ZERO_ on *power*.

Previous findings [15,43] suggest that during the bench press exercise, a higher load generally leads to increased EMG amplitude in the prime movers. However, this relationship is influenced by factors such as execution speed and the type of resistance employed. In this study, higher *load* did not affect EMG_PM_ amplitude during the first half of the ROM, and required the pectoralis major muscle to remain active for a longer duration, elevating EMG_PM_ only in the second half of the ROM (Figure 5). A similar pattern was observed in the anterior deltoid (Figure 7). Notably, the triceps brachii showed increased activity in the final 85% of the ROM as load increased (Figure 6). These results align well with previous findings [44]. Participants in this study were instructed to maximally accelerate the handles of the exercise apparatus, regardless of load or inertial resistance profile. The handles were constrained to move in the transverse plane along a convergent circular arc trajectory. Accordingly, the activity of the shoulder transverse flexors (e.g., the pectoralis major and anterior deltoid) remained nearly maximal as the handles were accelerated in the initial part of the movement, irrespective of load or inertial condition. In contrast, it is well established that the contribution of the triceps brachii is negligible at the beginning of the movement, and progressively increases during the concentric phase, peaking at the movement’s closure [44]. As such, the triceps brachii plays a negligible role in generating the initial burst of acceleration during the explosive exercise. This explains why the involvement of the triceps brachii increased proportionally with the resistance as soon as its contribution to the movement became significant. It also explains the lower EMG_TB_ amplitude observed under the I_FULL_ condition at the very end of the ROM. As previously discussed, in the final portion of the ROM, when the movement necessarily begins to decelerate, users must reduce the applied force to allow the full inertial resistance to decelerate to a stop as the elbows reach full extension. Consequently, this decreased force requirement translated into reduced effort from the triceps brachii, the most effective muscle for the movement closure, leading to a decrease in EMG_TB_ amplitude.

Maximum dynamic strength, rate of force development, and high-velocity strength —the ability to exert force at high contraction velocities—are fundamental individual neuromuscular components involved in the expression of explosive power [13]. “Mixed methods” training strategies employing a range of training modalities are routinely applied to develop each of these components with specific training stimuli and maximize explosive power capabilities [12,13]. The findings of the present study suggest that resistance with variable inertia (I_VAR_), generated by a robotic machine, constitutes a novel training modality that could enable the simultaneous development of these key power components. This advanced technology can generate inertial resistance (I_FULL_) during the acceleration phase of explosive movements, maximizing force production and RFD, and non-inertial resistance (I_ZERO_) in the final phase to minimize the decelerating portion of the ROM, thereby maximizing the high-velocity strength stimulus. As a result, I_VAR_ demonstrated superior performance to both I_FULL_ and I_ZERO_ in terms of force and power. The new method aims to replicate the neuromuscular patterns of ballistic exercises that are executed by releasing a gravitational implement in space to completely avoid the deceleration phase. While variable-inertia training with robotic strength machines cannot completely eliminate the final deceleration phase, it avoids the time- and space-consuming constraints and safety concerns associated with ballistic exercises [9].

The main limitation of this study lies in the relatively small sample size, with only ten participants recruited. This choice was dictated by several compelling factors: the need for participants with advanced experience in explosive resistance training, the demanding and complex experimental protocol, and, most importantly, the labor-intensive statistical analysis required for each time series to identify regions of the ROM showing significant differences between conditions (load and inertial profile). Given these constraints, a study with a larger sample would have been impractical, making a more focused investigation the most viable approach. Nevertheless, for each dependent variable (*force*, *velocity*, *power*, EMG_PM_, EMG_TB_, EMG_DA_), statistical power exceeded 0.55 in all ROM portions where significant differences were observed due to load or inertial profile variations. At the same time, a larger sample size would have reduced the likelihood of a Type II error, potentially increasing the number or extent of ROM portions exhibiting statistically significant differences. However, it cannot be ruled out that a larger sample could introduce greater interindividual variability, potentially reducing the statistical significance of some effects. This is precisely why we recruited participants with similar advanced experience in resistance training, ensuring a more homogeneous sample. Accordingly, the consistency of the present findings with the theoretical model proposed in [9] supports the rationale for further research aimed at expanding the evidence on the application of variable inertia profiles in resistance training.

This study employed an apparatus driven by a programmable electromechanical motor capable of generating novel resistances with an acceleration-dependent inertial component ($m\ddot{x}$) modulable within the ROM ($\lambda \left(x\right)\cdot m\ddot{x}$) independently of level its constant component (mg), i.e., the load level (see Equation (3)). Thus, a variety of distinct variable inertial profiles can, in principle, be programmed, in addition to the one (I_VAR_) considered. The analyzed I_VAR_ profile (Equation (6)) was selected for its potential in optimizing the mechanical output of explosive exercise, based on previous theoretical calculations [9]. Nevertheless, this profile could be potentially further refined to provide additional improvement on the exercise outcomes.

A final limitation concerns the residual inertia of the moving equipment elements (levers, pulleys, and cables), which cannot be completely eliminated as would be desirable for I_ZERO_ and in the last 25% of the ROM for Ivar. These undesired effects, although limited, could be further mitigated with equipment specifically designed for explosive exercises.

Further research employing larger sample sizes, comparisons of multiple variable inertia profiles, specialized equipment, and the analysis of long-term adaptations is needed to gain additional insight into the variable inertia training paradigm and its practical applications.

A final issue concerns the possible conflict of interest between two authors and Technogym S.p.A. (Cesena, Italy), the company where they work and which manufactures the Biostrength machines. Silvano Zanuso and Luca Zoffoli, as employees of Technogym S.p.A., contributed to the development of the Biostrength machines currently available on the market. However, the variable inertia resistance paradigm analyzed in this study was originally theorized in a previous work by Biscarini and Contemori [9] and is not implemented in the commercially available Biostrength machines. Indeed, its optimization would require the design of dedicated equipment with negligible residual inertia. Furthermore, Technogym S.p.A. had no role in the study design, data collection, analysis, or interpretation, nor in the writing of the manuscript or the decision to publish the results.

## 5. Conclusions

This study examined the effects of resistances with distinct inertial profiles (I_FULL_, I_ZERO_ and I_VAR_) on *force*, *power*, *velocity* and EMG amplitudes of the pectoralis major (EMG_PM_), triceps brachii (EMG_TB_), and anterior deltoid (EMG_DA_) during a chest press explosive exercise performed at varying intensity levels. These resistance profiles were generated by a programmable electromechanical motor integrated into a chest press machine. Overall, *I_ZERO_* facilitated faster movements but resulted in lower *force* outcomes, whereas *I_VAR_* produced higher *force* and *power* output. Higher loads increased EMG_PM_ and EMG_DA_ only during the second half of the concentric phase, following the initial burst of acceleration. *I_FULL_* reduced EMG_TB_ during the final portion of the concentric phase, with minimal impact on EMG_PM_ and EMG_DA_. This reflects the necessity of decelerating the inertial resistance near the end of the ROM and the critical role of the triceps brachii in finalizing the movement. The results of this study highlight the potential of resistances with variable inertia for enhancing force and power development through explosive exercises. More broadly, the modulation of inertia enabled by advanced electromechanical technologies offers an effective means of tailoring resistance exercises to optimize force, power, velocity, and muscle activation.

Machines equipped with programmable electromechanical motors are becoming increasingly common in both fitness and rehabilitation centers. While current costs remain relatively high, they are expected to decrease as these technologies become more widespread. Additionally, control software and digital interfaces can be easily adapted to facilitate seamless management of hybrid resistances with variable inertia, making these systems more accessible and user-friendly. Coaches and therapists are encouraged to adopt these innovative technologies and training paradigms to design more effective exercise interventions for athletes and patients.

## Figures and Tables

**Table 1:** Comparison of *load* and *inertia* across significant regions of the ROM.

Parameter	Effect	Region	Condition	Mean (95% CI)	
EMG_DA_ (%)	Load	ROM_0–4%_	1RM_30_^<1RM30, <1RM70^	69.26	(64.65–73.86)
			1RM_50_	78.00	(73.16–82.84)
			1RM_70_	80.62	(75.50–85.74)
		ROM_50–94%_	1RM_30_^<1RM30, <1RM70^	44.93	(39.60–50.26)
			1RM_50_^<1RM70^	55.37	(51.54–59.20)
			1RM_70_	80.62	(75.50–85.74)
EMG_PM_ (%)	Load	ROM_50–97%_	1RM_30_^<1RM30,<1RM70^	41.22	(36.98–45.47)
			1RM_50_^<1RM70^	50.22	(46.51–53.93)
			1RM_70_	62.46	(58.75–66.17)
EMG_TB_ (%)	Inertia	ROM_98–100%_	I_FULL_^<IVAR,<IZERO^	21.32	(17.83–24.80)
			I_VAR_	29.31	(25.18–33.44)
			I_ZERO_	29.07	(25.06–33.08)
	Load	ROM_13–97%_	1RM_30_^<1RM30,<1RM70^	61.90	(57.54–66.26)
			1RM_50_^<1RM70^	72.33	(67.48–77.18)
			1RM_70_	83.14	(77.81–88.48)
Force (kgf)	Inertia	ROM_18–79%_	I_FULL_^<IVAR^	53.12	(49.71–56.52)
			I_VAR_	55.31	(52.10–58.52)
			I_ZERO_^<IFULL,<IVAR^	47.82	(44.35–51.30)
	Load	ROM_50–94%_	1RM_30_^<1RM30,<1RM70^	39.32	(36.98–41.66)
			1RM_50_^<1RM70^	49.62	(47.21–51.63)
			1RM_70_	60.04	(56.64–63.44)
Power (W)	Inertia	ROM_80–100%_	I_FULL_^<IVAR,<IZERO^	275.2	(241.3–309.2)
			I_VAR_	376.2	(346.3–406.2)
			I_ZERO_^<IVAR^	325.3	(293.5–357.1)
	Load	ROM_15–62%_	1RM_30_	628.7	(591.1–666.3)
			1RM_50_	607.4	(579.1–635.8)
			1RM_70_^<1RM30,<1RM50^	462.5	(439.7–485.2)
		ROM_93–100%_	1RM_30_^<1RM50,<1RM70^	124.4	(102.4–146.3)
			1RM_50_^<1RM70^	194.7	(173.7–215.6)
			1RM_70_	222.5	(203.8–241.2)
Velocity (cm/s)	Inertia	ROM_3–100%_	I_FULL_^<IVAR,<IZERO^	96.09	(90.31–101.8)
			I_VAR_^<IZERO^	99.59	(93.22–105.9)
			I_ZERO_	112.5	(106.5–118.6)
	Load	ROM_0–96%_	1RM_30_	125.9	(123.0–128.8)
			1RM_50_^<1RM30^	107.8	(104.0–111.7)
			1RM_70_^<1RM30,<1RM50^	75.19	(71.42–78.97)

## Data Availability

The raw data supporting the conclusions of this article will be made available by the authors on request.

## Abbreviations

The following abbreviations are used in this manuscript:

ROM	Range of motion
ROM%	Instantaneous position of the resistance levers, expressed as a percentage (0–100%) of the individual range of motion determined by the exercise apparatus for each participant.
1RM	One-repetition maximum
EMG	Electromyography/Electromyographic
